# Association Between Anxiety Symptoms and Problematic Smartphone Use Among Chinese University Students: The Mediating/Moderating Role of Self-Efficacy

**DOI:** 10.3389/fpsyt.2021.581367

**Published:** 2021-02-24

**Authors:** Ying Li, Guang-Xiao Li, Ming-Li Yu, Chun-Li Liu, Yun-Ting Qu, Hui Wu

**Affiliations:** ^1^Department of Social Medicine, School of Public Health, China Medical University, Shenyang, China; ^2^Medical Record Management Center, The First Affiliated Hospital of China Medical University, Shenyang, China; ^3^School of Medical Information, China Medical University, Shenyang, China

**Keywords:** University students, mediator, moderator, self-efficacy, anxiety symptoms, problematic smartphone use

## Abstract

Problematic smartphone use (PSU) is a novel manifestation of addictive behaviors. It is frequently reported to be correlated with anxiety symptoms among University students. However, the underlying mechanism has not yet been thoroughly studied. Whether the association between anxiety symptoms and PSU is mediated or moderated by self-efficacy remains unclarified. A cluster sampling cross-sectional study was thus conducted to explore the potential mediating or moderating effect of self-efficacy in Chinese University students. Participants (*N* = 1,113) were recruited from eight Universities in Shenyang, China. Of them, 146 did not effectively respond to the questionnaires. Thus, 967 participants were eligible for the final analysis. The mediating or moderating role of self-efficacy in the anxiety-PSU relationship was explored using hierarchical multiple regression. Then the mediation model was further verified using the SPSS macros program (PROCESS v3.0). Our results showed that anxiety symptoms was positively correlated with PSU (*r* = 0.302, *P* < 0.01), while self-efficacy was negatively correlated with anxiety symptoms and PSU (*r* = −0.271 and −0.181, *P* < 0.01). Self-efficacy partly mediated the relationship between anxiety symptoms and PSU, which accounted for ~17.5% of the total effect that anxiety symptoms have on PSU. However, the moderating effect of self-efficacy on the anxiety-PSU relationship was insignificant. In summary, our findings suggested that self-efficacy partly mediates but not moderates the link between anxiety symptoms and PSU among Chinese University students. Therefore, multicomponent interventions should be made to restrict the frequency of smartphone usage, enhance the level of self-efficacy, and thus promote the mental health status of University students.

## Introduction

### Background

A smartphone is no longer simply considered a “mobile phone” but rather a portable and omnipotent pocket computer. Owning smartphones enables us to keep in touch with our friends anywhere at any time, helps us to stay organized, guarantee stress-free travel through navigation apps, helps us to cope with emergencies, offers easy access to information and technology, and even promotes health through health-related apps (1). Given the convenience that smartphones provide to our daily lives, they have become pervasively used globally. According to a recent mobile user statistic report (2), the number of global smartphone users has reached 3.5 billion, increasing by 40% from 2016 to 2020. Therefore, smartphones have long been unconsciously and closely integrated into people's daily lives and gradually changed our lifestyles.

However, smartphone usage is a double-edged sword, as it helps to facilitate our daily lives but might also cause a series of worrisome problems due to problematic smartphone use (PSU). PSU has been previously defined as excessive use of a smartphone that is accompanied by functional impairments in daily living, and substance addiction-like symptoms (3). Youths, especially University students, are digital natives and the fastest adopters of electronic technologies (4). Unfortunately, they are usually mentally immature and lack the self-regulatory ability (5). Therefore, they are more vulnerable to PSU than older adults. It was demonstrated that PSU can have many detrimental effects, as it can cause academic distractions (6, 7), physical health hazards (including wrist pain, neck disability, and vision impairment) (8–10), and as well as elevated accident risk (11). Additionally, accumulating evidence has shown that PSU is closely related to poor mental health, particularly depression, anxiety, and perceived stress (12–15).

Anxiety is one of the most commonly investigated mental health variables related to PSU (15–17). A review by Elhai et al. revealed a small-to-moderate positive association between anxiety and PSU (15). A higher degree of anxiety symptoms was positively associated with more severe PSU. The prevalence of anxiety symptoms among University students with PSU was ~1.78- to 2.31-fold higher than that among those without PSU (16). Several theoretical frameworks have been developed to help explain how psychological and psychopathological constructs such as anxiety could relate to PSU (17). The uses and gratifications theory (UGT) (18) proposes motivations (including psychological characteristics) for media usage. Based on the UGT, anxiety can drive people to use or overuse smartphones so as to satisfy or calm their anxiety. Another theoretical model that is more specific to psychopathological constructs is the compensatory Internet use theory (CIUT) (19). The CIUT assumes that excessive internet use, such as PSU, resulted from an attempt to alleviate negative emotions after experiencing stressful life events. A more plausible theoretical framework is the Interaction of Person-Affect-Cognition-Execution (I-PACE) (20, 21). Initially, the I-PACE model conceptualized personal background and predisposing factors such as anxiety symptoms as an important influence of problematic Internet use (PIU). The predisposing factors might cause an affective/cognitive response, and the latter also has a substantial impact on PSU. Under the framework of the I-PACE model, affective and cognitive response variables are usually conceptualized as mediators/moderators explaining the relationships between predisposing factors and PIU (20, 21).

Self-efficacy refers to individuals' beliefs in their own capabilities to execute behaviors necessary to produce specific performances (22). Belief in self-efficacy may have some impacts on an individual's cognitions, affects, and behaviors and may also help to deal with stressful situations (22). Evidence has shown that self-efficacy is negatively correlated with anxiety symptoms (23). A low level of self-efficacy is usually accompanied by a high level of anxiety symptoms. Similarly, a low level of self-efficacy was associated with a higher level of PSU severity (24, 25). According to the I-PACE model, self-efficacy could be regarded as a cognitive component; thus, it was reasonable to conceptualize self-efficacy as a potential mediating or moderating variable in the model. Actually, the mediating and buffering effect of self-efficacy on the relationship between PSU and other psychological variables such as academic procrastination and materialism has been previously reported (24, 25). Although numerous studies support the relationship between PSU and anxiety symptoms (17), whether self-efficacy can serve as a mediator or moderator on this relationship remains unknown.

### Aims

Our primary aim was to clarify the role of self-efficacy in explaining the relationship between anxiety symptoms and the severity of PSU based on a sample of Chinese University students. We were particularly interested in the mediating and moderating effect of self-efficacy.

### Theory

The most widely accepted theoretical framework underlying the PIU or PSU is the I-PACE model (17, 20, 21, 26–30). I-PACE proposes several categories of variables that influence excessive internet use. The first category includes personal predisposing variables such as personality, psychopathology, and internet use motive-based influences (28). The second category involves affective and cognitive response variables consisting of coping strategy, attention bias, mood dysregulation, and responses to environmental stressors (28). These response variables are usually conceptualized as mediators and moderators for the relationship between personal predisposing variables and PIU or PSU (20). Last, the I-PACE model assumes that response variables may have some impact on a person's decisions regarding a particular pattern of internet use, and thus may result in adaptive, problematic use. Based on the I-PACE model, anxiety-related psychopathology is what drives PSU, rather than the other way around. Self-efficacy fits well with the cognitive processes (or biases) in the affective and cognitive response variable category of the I-PACE model (20, 21). Therefore, it is reasonable to hypothesize that self-efficacy should mediate or moderate the anxiety-PSU relationship.

### Hypotheses

H1. *Anxiety symptoms severity should be positively correlated with the severity of PSU*. The association between anxiety symptoms and PSU severity has been previously confirmed by many studies (15, 17, 27). Anxiety could be regarded as one of the individual's predisposing variables of the I-PACE model that can cause PSU (20, 21).

H2. *Self-efficacy should be negatively correlated with the severity of anxiety symptoms*. A low level of self-efficacy will lead to poor management of negative life events and thus result in anxiety symptoms (23).

H3. *Self-efficacy should be negatively correlated with PSU severity*. Several previous studies from Asia (24, 25, 31) support self-efficacy's negative relationship with PSU severity.

H4: *Self-efficacy should mediate the association between anxiety symptoms and PSU severity*. Self-efficacy can be considered as one of the affective and cognitive response variables in the I-PACE model (20, 21). A Korean study found that the relationship between depression and PSU could be fully mediated by self-efficacy (32).

H5. *Self-efficacy should moderate the association between anxiety symptoms and PSU severity*. As a potential affective and cognitive response variable in the I-PACE model, self-efficacy might moderate the association between anxiety symptoms and PSU as well.

## Materials and Methods

### Study Design and Data Collection

**Figure 1:**
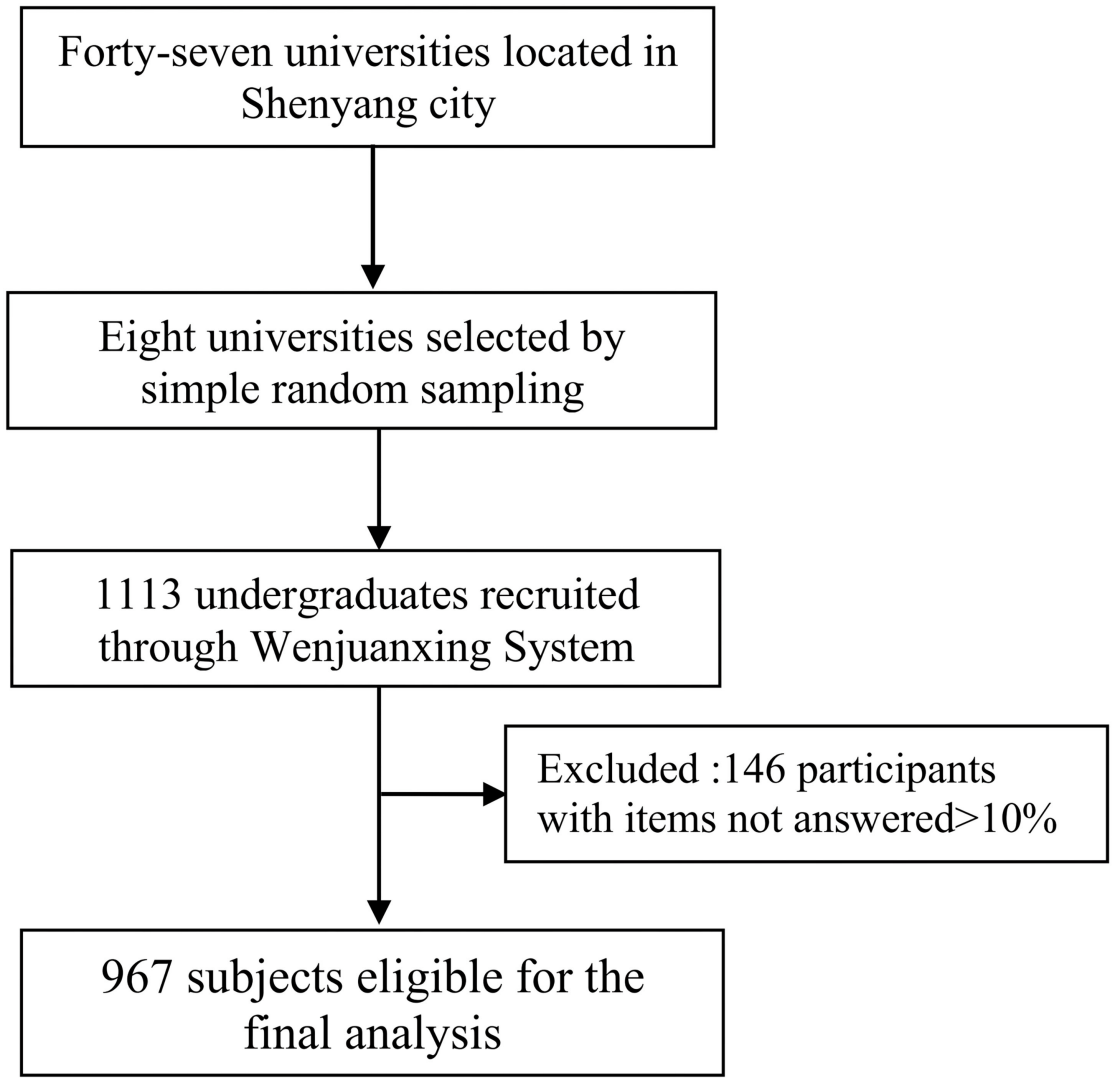
Flow chart for participant selection.

The current study was a school-based cross-sectional study using a cluster random sampling strategy. From November 2018 to March 2019, students from eight universities were randomly selected from Shenyang city located in northeastern China. Participants came from medical universities, normal universities, and other majors. Their participation in our survey was voluntary, and they were free to withdraw at any time without being forced to complete the tasks. Electronic informed consent was obtained from each participant before the investigation, and then all participants were asked to answer self-rating questionnaires using the Wenjuanxing Online Survey System (https://www.wjx.cn/). Finally, a total of 1,113 undergraduate students were then randomly recruited. Of them, 146 were excluded due to incomplete responses, with at least 10% of the items not answered. Thus, only 967 subjects were eligible for the final analysis, resulting in an effective response rate of 86.9%. Figure 1 showed the process of participant selection. Information obtained from all participants was ensured confidential and anonymous at all times. The study protocol was consistent with the ethical standards and was approved by the Ethics Committee of China Medical University.

### Measures of Anxiety Symptoms

The Chinese version of the 20-item Self-Rating Anxiety Scale (SAS) developed by Zung was employed to assess the level of anxiety symptoms among University students during the past week (33). The SAS consists of 20 items that are rated on a 4-point Likert-like scale ranging from “1 = none or a little of the time (<1 day)” to “4 = most or all the time (5–7 days).” Of these items, 5, 9, 13, 17, and 19 items are reverse scoring questions. The summative score is obtained by multiplying the total score by 1.25 and taking an integer. Higher scores indicate more severe anxiety symptoms. The SAS scale has been proven reliable and of good internal consistency among the Chinese population (33, 34), with a Cronbach's alpha coe?cient of 0.806 in the current study.

### Measures of Smartphone Addiction

The Smartphone Addiction Scale-Short Version (SAS-SV) (35) was employed to measure the severity of PSU. This self-rating scale contains 10 negative items. Each item is rated on a six-point Likert-type scale ranging from “1 = Strongly disagree” to “6 = Strongly agree” to reflect smartphone usage during the past month. Higher scores represent a higher risk of PSU. The total score ranged from 10 to 60. The Chinese version of the SAS-SV has been confirmed to have good reliability and validity (36). The Cronbach's alpha in our sample was 0.893.

### Measures of Self-Efficacy

Self-efficacy was assessed using the General Self-Efficacy Scale (GSES) designed by Schwarzer et al. (37). The self-reported scale measures the degree of one's belief concerning ability and persistence to achieve the required performances. The questionnaire consists of 10 positive items, and the option for each item is scored according to a 4-point Likert scale from “1= strongly disagree” to “4=strongly agree.” The theoretical score of the scale ranges from 0 to 40 points. Higher total scores indicate higher levels of self-efficacy. GSE scale has been widely used internationally. The empirical literature has shown that the Chinese version of the GSE also has good reliability and validity when applied to the Chinese population (38, 39). Cronbach's alpha was 0.818 in our study.

### Statistical Analysis

All statistical analyses were performed using IBM SPSS Statistics for Windows (version 23.0; IBM Corp., Asia Analytics Shanghai). All tests were single-tailed, with *P* < 0.05 considered statistically significant. The group differences of continuous variables were tested by *t*-test or ANOVA as appropriate. The *post-hoc* analysis for multiple comparisons was conducted using Dunnett's *t-*test. A correlation matrix was examined using Pearson's correlation analysis among PSU, self-efficacy, and anxiety symptoms. Missing values were imputed by multiple imputation method.

The mediating or moderating role of self-efficacy in the anxiety-PSU relationship was explored using hierarchical multiple regression. All demographic variables that were significantly associated with PSU by univariate linear regression analysis served as control covariates. Category variables were transformed into dummy variables. Furthermore, continuous independent variables and mediator/moderator (self-efficacy) were centralized before hierarchical multiple regression. The covariates, independent variables, and mediator/moderator were sequentially included in the regression models in three steps. To explore the potential mediating role of self-efficacy, the covariates were added in step 1; anxiety symptoms was added as the independent variable in step 2; self-efficacy was added as a mediator in step 3. Similarly, to explore the potential moderating role of self-efficacy, the covariates were added 1 in step 1. However, in step 2, both anxiety symptoms and self-efficacy were included. The product of anxiety symptoms and self-efficacy was added in step 3. Multicollinearity was examined by the variance inflation factor (VIF). A VIF value>10 indicated the existence of a serious multicollinear problem.

The potential mediating role of self-efficacy was further verified using the SPSS macros program (PROCESS v3.0 by Andrew F. Hayes) with 5,000 bootstrap sampling (40). The control covariates were the same as those used in the hierarchical multiple regression. Anxiety symptoms was treated as the independent variable, with PSU as the dependent variable, and self-efficacy as the mediator. Their total scores were standardized separately to eliminate the differences in scale scores. The total effect (path c), the direct effect (path c′), and the indirect effects (path a^*^b) were checked. The bias-corrected and accelerated 95% confidence interval (BCa 95%CI) for the indirect effect was also calculated. The mediating effect is considered statistically significant if the 95%CI of indirect effect (path a^*^c) does not contain zero.

## Results

### Demographic Characteristics

The demographic characteristics of the study participants and group differences are shown in Table 1.

**Table 1:** Demographic characteristics of the study participants (*N* = 967) and univariate analysis for the factors related to the level of PSU, self-efficacy, and anxiety symptoms.

**Variables**	**Number (%)**	**PSU**	**Self-efficacy**	**Anxiety symptoms**
		**(Mean ± SD)**	**(Mean ± SD)**	**(Mean ± SD)**
**Sex**				
Male	490 (50.7)	37.89 ± 8.57	26.20 ± 5.12	52.37 ± 11.16
Female	477 (49.3)	39.65 ± 9.25^**^	26.34 ± 5.10	53.89 ± 9.89^*^
**Grade**				
Freshman	204 (21.1)	34.31 ± 8.50	25.06 ± 5.39	51.74 ± 10.91
Sophomore	249 (25.7)	37.43 ± 8.24^**^	25.44 ± 5.00	53.59 ± 12.12
Junior	415 (42.9)	41.32 ± 8.64^**^	27.18 ± 4.72^**^	52.79 ± 8.82
Senior	99 (10.2)	40.52 ± 8.79^**^	27.00 ± 5.59^**^	56.17 ± 11.91^**^
**Monthly living expenses (yuan)**				
<1,000	88 (9.1)	35.06 ± 7.61	24.62 ± 5.91	52.87 ± 12.88
1,000–3,000	671 (69.4)	39.46 ± 9.09^**^	26.33 ± 4.89^**^	52.86 ± 10.00
>3,000	208 (21.5)	38.07 ± 8.62^*^	26.76 ± 5.34^**^	54.06 ± 11.40
**Residential area**				
Urban	479 (49.5)	37.99 ± 9.02	26.06 ± 5.20	53.20 ± 10.73
Rural	488 (50.5)	39.51 ± 8.83^**^	26.47 ± 5.02	53.05 ± 10.43
**Whether or not the only child**				
Yes	546 (56.5)	38.44 ± 8.95	26.15 ± 5.11	54.06 ± 11.03
No	421 (43.5)	39.17 ± 8.96	26.41 ± 5.11	51.90 ± 9.85^**^

*Missing values: grade, 4 cases; monthly living expenses, 5 cases; whether or not the only child, 4 cases; PSU, 2 cases; self-efficacy, 4 cases; anxiety symptoms, 3 cases*.

* *P < 0.05*,

** *P < 0.01*.

Female University students had higher PSU and anxiety symptoms than male University students (PSU: 39.65 vs. 37.89, *P* < 0.01; anxiety symptoms: 53.89 vs. 52.37, *P* < 0.05), but no sex difference was found regards to self-efficacy. The levels of PSU, self-efficacy, and anxiety symptoms significantly varied across the different grades of University students (*P* < 0.01). Monthly living expenses higher than 1,000 yuan were associated with elevated levels of PSU and self-efficacy, but not with anxiety symptoms. The PSU scores were significantly different between students from urban and rural areas (37.99 vs. 39.51, *P* < 0.01). Finally, University students who had no siblings tended to have higher levels of anxiety disorders than those who had siblings (54.06 vs. 51.90, *P* < 0.01).

### Correlations Among PSU, Self-Efficacy, and Anxiety Symptoms

The mean values and bivariate correlations between continuous variables are shown in Table 2. Anxiety symptoms was positively correlated with PSU (*r* = 0.302, *P* < 0.01). In contrast, self-efficacy was negatively associated with both anxiety symptoms and PSU (*r* = −0.271 and −0.181, *P* < 0.01).

**Table 2:** The means, standard deviations, and bivariate correlations between continuous variables.

**Variables**	**(Mean ± SD)**	**1**	**2**	**3**
1. Age	20.36 ± 1.50	1		
2. Anxiety symptoms	53.12 ± 10.58	0.084^**^	1	
3. PSU	38.74 ± 8.94	0.148^**^	0.302^**^	1
4. Self-efficacy	26.27 ± 5.10	0.100^**^	−0.271^**^	−0.181^**^

*PSU, problematic smartphone use; SD, standard deviation*.

** *P < 0.01*.

### Mediating Effect of Self-Efficacy on the Relationship Between Anxiety Symptoms and PSU Severity

As shown in Table 3, the control covariates in step 1 significantly explained PSU (adjusted *R*^2^ = 0.113, Δ*R*^2^ = 0.120, *P* < 0.01). Among them, age, sex, grade, and monthly living expense were significantly related to PSU severity. In step 2, after adjusting for control covariates, anxiety symptoms was positively associated with PSU (β = 0.292, *P* < 0.01). Anxiety symptoms explained additional 8.3% of the variance of PSU. In step 3, self-efficacy was negatively associated with PSU (β = −0.181, *P* < 0.01), which accounted for additional 2.9% of the variance. When self-efficacy was added to the model, the absolute value of the regression coefficient of anxiety symptoms on PSU was decreased from 0.292 to 0.241. Therefore, self-efficacy might probably serve as a mediator in the association between anxiety symptoms and PSU among University students.

**Table 3:** The mediating effect of self-efficacy on the relationship between anxiety symptoms and PSU severity among University students.

**Variables**	**Block 1**		**Block 2**		**Block 3**	
	**β**	**VIFs**	**β**	**VIFs**	**β**	**VIFs**
**Step 1**						
Age	−0.128^**^	2.502	−0.148^**^	2.507	−0.159^**^	2.511
Female vs. male	0.063^*^	1.034	0.040	1.041	0.042	1.041
Rural vs. urban	0.035	1.154	0.038	1.154	0.044	1.155
Grade 2 vs. grade 1	0.191^**^	1.910	0.174^**^	1.914	0.187^**^	1.919
Grade 3 vs. grade 1	0.460^**^	3.404	0.463^**^	3.404	0.508^**^	3.472
Grade 4 vs. grade 1	0.304 ^**^	2.683	0.282^**^	2.688	0.314^**^	2.722
Monthly living expenses (yuan)						
1,000–3,000 vs. <1,000	0.117^*^	2.838	0.117^*^	2.838	0.133^**^	2.847
>3,000 vs. <1,000	0.057	2.981	0.048	2.982	0.073	3.003
**Step 2**						
Anxiety symptoms			0.292^**^	1.022	0.241^**^	1.113
**Step 3**						
Self-efficacy					−0.181^**^	1.137
*F*	16.351^**^		100.202^**^		36.049	
Adjusted *R^2^*	0.113		0.196		0.224	
Δ*R^2^*	0.120		0.083		0.029	

*Grade 1. Freshman; Grade 2, Sophomore; Grade 3, Junior; Grade 4, Senior; PSU, problematic smartphone use; VIF, Variance inflation factor*.

* *P < 0.05*;

** *P < 0.01*.

**Figure 2:**
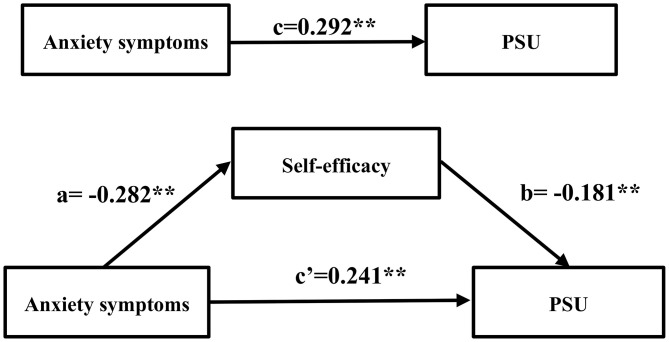
Model of the mediating role of self-efficacy between anxiety symptoms and PSU severity. **P < 0.01.

Regarding the implications of hierarchical multiple regression, the mediation of self-efficacy in the anxiety-PSU relationship was further validated using PROCESS v 3.0. Table 4 demonstrated the results of the mediation analysis. First, the association between anxiety symptoms and PSU (c path) was calculated. Anxiety symptoms was positively associated with PSU (*c* = 0.292, *P* < 0.01). Second, the indirect effect of anxiety symptoms on PSU via self-efficacy was found statistically significant [path a^*^b, a = −0.282, b = −0.181, a^*^b(BCa 95%CI) = 0.051(0.029, 0.075)]. Since the confidence interval for indirect effect did not include the null value, then we could conclude that self-efficacy played a mediating role between anxiety symptoms and PSU. Finally, when self-efficacy was included in the model as a mediator, the direct effect of anxiety symptoms on PSU (path c′) remained statistically significant (c′ = −0.241, *P* < 0.01). Therefore, self-efficacy had a partial mediating effect on the association between anxiety symptoms and PSU for University students. The mediation of self-efficacy accounted for ~17.5% (a^*^b/c) of the total effect. The visualization of the model was demonstrated in Figure 2.

**Table 4:** The results of the mediation analysis Path Coefficient/Effect *P*-value BCa 95% CI.

**Path**	**Coefficient/effect**	***P*-value**	**BCa 95%CI**
c	0.292	<0.01	(0.235, 0.349)
a	−0.282	<0.01	(−0.342, −0.222)
b	−0.181	<0.01	(−0.241, −0.122)
a*b	0.051	-	(0.029, 0.075)
c′	0.241	<0.01	(0.182, 0.300)

*BCa 95% CI the bias-corrected and accelerated 95% confidence interval; Age, gender, location, grade, monthly living expenses were covariates*.

### Moderating Effect of Self-Efficacy on the Relationship Between Anxiety Symptoms and PSU Severity

As shown in Table 5, in step 2, anxiety symptoms was positively associated with PSU after the adjustment of control variables (β = 0.241, *P* < 0.01), while self-efficacy was negatively associated with PSU (β = −0.181, *P* < 0.01). The model fits were significantly improved by anxiety symptoms and self-efficacy (adjusted *R*^2^ = 0.224, Δ*R*^2^= 0.112, *P* < 0.01). In step 3, the interaction term of anxiety symptoms and self-efficacy was not statistically significant (β = 0.003, *P* = 0.98). Thus, self-efficacy could not moderate the relationship between anxiety symptoms and PSU among University students.

**Table 5:** The moderating effect of self-efficacy on the relationship between anxiety symptoms and PSU severity among University students.

**Variables**	**Block 1**		**Block 2**		**Block 3**	
	**β**	**VIFs**	**β**	**VIFs**	**β**	**VIFs**
**Step 1**						
Age	−0.128^**^	2.502	−0.159^**^	2.511	−0.159^**^	2.511
Female vs. male	0.063^*^	1.034	0.042	1.041	0.042	1.043
Rural vs. urban	0.035	1.154	0.044	1.155	0.043	1.155
Grade 2 vs. grade 1	0.191^**^	1.910	0.187^**^	1.919	0.186^**^	1.922
Grade 3 vs. grade 1	0.460^**^	3.404	0.508^**^	3.472	0.507^**^	3.479
Grade 4 vs. grade 1	0.304 ^**^	2.683	0.314^**^	2.722	0.314^**^	2.722
Monthly living expenses (yuan)						
1,000–3,000 vs. <1,000	0.117^*^	2.838	0.133^**^	2.847	0.133^**^	2.849
>3,000 vs. <1,000	0.057	2.981	0.073	3.003	0.074	3.006
**Step 2**						
Anxiety symptoms			0.241^**^	1.113	0.241^**^	1.122
Self-efficacy			−0.181^**^	1.137	−0.181^**^	1.159
**Step 3**						
Interaction item					0.003	1.032
*F*	16.351^**^		69.961^**^		0.013	
Adjusted *R^2^*	0.113		0.224		0.224	
Δ*R^2^*	0.120		0.112		0	

*Grade 1, Freshman; Grade 2, Sophomore; Grade 3, Junior; Grade 4, Senior; PSU, problematic smartphone use; VIF, Variance inflation factor*;

* *P < 0.05*;

** *P < 0.01*.

## Discussion

### Main Findings

The main findings of this study were as follows. A higher level of anxiety symptoms was significantly correlated with more severe PSU. There was a significantly negative association between self-efficacy and anxiety symptoms. Furthermore, self-efficacy can mediate the association between anxiety symptoms and PSU. Nevertheless, the moderating effect of self-efficacy on the association between anxiety symptoms and PSU was insignificant. To the best of our knowledge, this was the first study to explore the mediating/moderating effect of self-efficacy on the relationship between anxiety symptoms and excessive smartphone use in University students.

Our study reported a small to moderate positive correlation between anxiety symptoms and PSU among University students, which was consistent with findings from previous empirical studies (1, 17). Moreover, hierarchical multiple regression analyses showed that a high level of anxiety symptoms was an independent predictor of severe PSU. Our findings could be explained by some theoretical frameworks. The UGT treats anxiety as a motivator that drives people to overuse smartphones to calm their anxiety (18). The CIUT proposes that PSU results from people's attempt to relieve their negative emotions from stressful life events (19). The I-PACE model regards anxiety symptoms as predisposing factors that have an important influence on PSU (20, 21).

As expected, we found a negative correlation between self-efficacy and anxiety symptoms in our study. Self-efficacy, as one of the most important positive psychological qualities, has become a plastic internal psychological resource and can serve as a buffer against mental disorders (41–43). CL Liu et al. demonstrated that self-efficacy was negatively correlated with the levels of both depression and anxiety among doctoral students (44). Self-efficacy training has been confirmed effective in reducing mental problems, such as anxiety and depression (45). Similarly, the negative correlation between self-efficacy and PSU was validated by our study. A previous study showed that a high level of self-efficacy might serve as a buffer to addiction-like behaviors such as problematic gambling, resulting in a weakened relationship (46). Additionally, randomized controlled trials by improving self-efficacy have been proven to be effective in the treatments of tobacco, alcohol, and drug addictions among college students (47, 48).

Our findings suggested that self-efficacy could act as a mediator between anxiety symptoms and PSU among University students. The mediating effect of self-efficacy could explain ~17.5% of the total effect that anxiety symptoms have on PSU. Nevertheless, the moderating effect of self-efficacy on the anxiety-PSU relationship was insignificant. As mentioned in the Introduction section, self-efficacy can be treated as one of the affective and cognitive response variables of the I-PACE model (20, 21). Therefore, it is not surprising that self-efficacy can mediate the relationship between anxiety symptoms and PSU. Similar findings were reported in a population of Korean nursing students (32). They found that self-efficacy could fully mediate the relationship between depression and PSU. The potential mediating mechanisms of self-efficacy in preventing and reducing other addictive behaviors such as Internet and gambling addictions have been previously reported (46, 49).

However, no studies have investigated the mediating or moderating role of self-efficacy in the anxiety-PSU relationship. We believe that students who perceive a higher level of self-efficacy usually possess more confidence and perseverance to cope with interpersonal troubles and have a higher level of self-control over their impulsivity to pursue pleasure through smartphones, resulting in decreased exposure to PSU compared to those with a lower level of self-efficacy (50). Meanwhile, self-efficacy, as a well-known positive psychological resource, can also help to reduce adverse anxiety psychological problems effectively (51). Given the mediating role of self-efficacy in our findings, this model should be applied to provide a possible framework for the development of health education and health-related inventions. Effective strategies should be taken to resist the psychological dependence of smartphone usage, improve the level of self-efficacy, and thus relieve mental health disorders among University students.

### Limitations

Several limitations should be taken into account in this study. First, data were collected at just one timepoint instead of longitudinally which limited the ability to establish the causal inferences or determine the direction of the causal relationships. Future prospective studies with a large sample size are warranted to validate our findings. Second, the survey was conducted using self-report questionnaires, which might not objectively reflect the actual smartphone usage and psychological exposures. Third, other psychiatric disorders such as depression personality disorders and medications for psychiatric reasons were not investigated, which would prevent us from fully understanding the mechanisms between various psychological factors and problematic smartphone use. Fourth, a sample of adolescent Chinese students may disable the external generalization of our findings. More representative samples of general smartphone users are needed. Finally, as an observational study, the mediating effect of self-efficacy on the relationship between anxiety and PSU should be confirmed by randomized controlled trials. Future research on interventions should be extensively conducted to verify our hypothetical models and radically prevent addictive behaviors among college students.

## Conclusion

PSU can cause many detrimental psychological disorders such as anxiety symptoms. It has become a mental health threat to University students. The current study is the first to provide evidence that self-efficacy can partly mediate the association between PSU and anxiety symptoms in Chinese University students. Considering the increasing prevalence of PSU among University students, multicomponent interventions, from the joint efforts of school-family-students, should be made to restrict the frequency of smartphone usage and increase the level of self-efficacy to thus promote the mental health of University students.

## Data Availability Statement

The raw data supporting the conclusions of this article will be made available by the authors, without undue reservation.

## Ethics Statement

The studies involving human participants were reviewed and approved by the Ethics Committee of China Medical University. The patients/participants provided their electronic informed consent to participate in this study.

## Author Contributions

YL and M-LY contributed to data collection, statistical analysis, and revision of the manuscript. YL drafted the manuscript. Y-TQ and C-LL contributed to organizing the survey and interpretation of the data. HW and G-XL contributed to the study design, data collection, and revision of the manuscript. All authors read and approved the final version of the manuscript for submission.

## Conflict of Interest

The authors declare that the research was conducted in the absence of any commercial or financial relationships that could be construed as a potential conflict of interest.
